# Permeability Barrier and Microstructure of Skin Lipid Membrane Models of Impaired Glucosylceramide Processing

**DOI:** 10.1038/s41598-017-06990-7

**Published:** 2017-07-25

**Authors:** Michaela Sochorová, Klára Staňková, Petra Pullmannová, Andrej Kováčik, Jarmila Zbytovská, Kateřina Vávrová

**Affiliations:** 1 Skin Barrier Research Group, Charles University, Faculty of Pharmacy, Hradec Králové, 500 05 Czech Republic; 2 Department of Pharmaceutical Technology, Faculty of Pharmacy, Hradec Králové, 500 05 Czech Republic; 3 Department of Organic Technology, University of Chemistry and Technology Prague, 166 28 Prague, Czech Republic

## Abstract

Ceramide (Cer) release from glucosylceramides (GlcCer) is critical for the formation of the skin permeability barrier. Changes in β-glucocerebrosidase (GlcCer’ase) activity lead to diminished Cer, GlcCer accumulation and structural defects in SC lipid lamellae; however, the molecular basis for this impairment is not clear. We investigated impaired GlcCer-to-Cer processing in human Cer membranes to determine the physicochemical properties responsible for the barrier defects. Minor impairment (5–25%) of the Cer generation from GlcCer decreased the permeability of the model membrane to four markers and altered the membrane microstructure (studied by X-ray powder diffraction and infrared spectroscopy), in agreement with the effects of topical GlcCer in human skin. At these concentrations, the accumulation of GlcCer was a stronger contributor to this disturbance than the lack of human Cer. However, replacement of 50–100% human Cer by GlcCer led to the formation of a new lamellar phase and the maintenance of a rather good barrier to the four studied permeability markers. These findings suggest that the major cause of the impaired water permeability barrier in complete GlcCer’ase deficiency is not the accumulation of free GlcCer but other factors, possibly the retention of GlcCer bound in the corneocyte lipid envelope.

## Introduction

The epidermal barrier protects the body from excessive water loss and the entry of exogenous substances. An essential component of the permeability barrier is the extracellular lipid matrix of the stratum corneum (SC), the uppermost epidermal layer. This lipid matrix consists of ceramides (Cer), free fatty acids (FFA) and cholesterol (Chol) with a minor amount of cholesteryl sulfate (CholS)^1, 2^. The most unusual but critical lipids that mediate the permeability barrier are Cer^3^.

**Figure 1:**
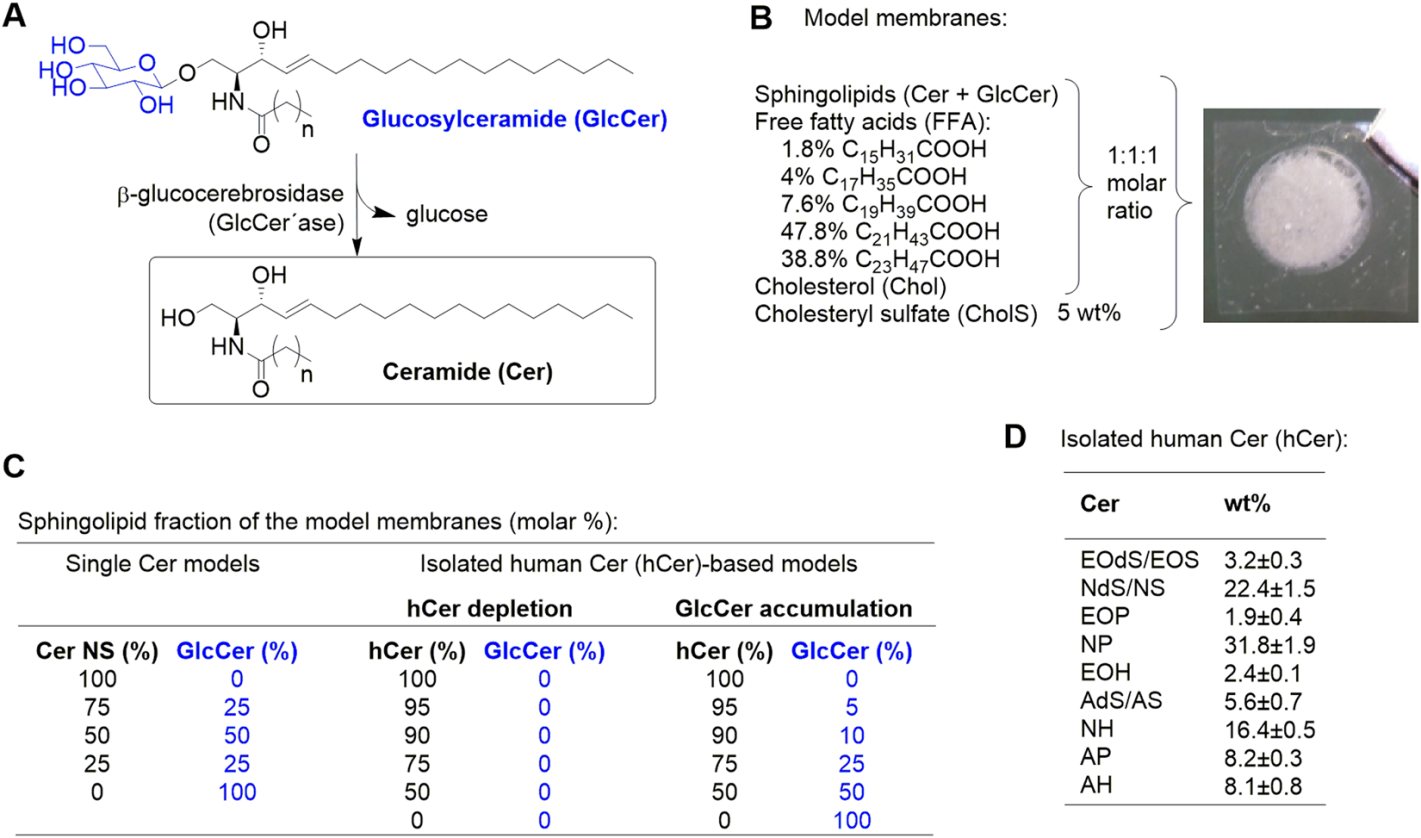
Scheme of the Conversion of Glucosylceramide (GlcCer) to Ceramide (Cer) by β-Glucocerebrosidase (GlcCer’ase) (**A**) and the Composition of the Model Membranes (**B**–**D**). The model membranes were constructed from sphingolipids (Cer and/or GlcCer, Chol and a mixture of FFA in an equimolar ratio with 5 wt% CholS (**B**,**C**). For single Cer membranes, *N*-tetracosanoyl D-*erythro*-sphingosine (CerNS) was used, whereas the complex model system was constructed from isolated human SC Cer (hCer). The composition of hCer is specified in panel D. To simulate deficient GlcCer-to-Cer processing, the Cer fraction was gradually diminished with or without GlcCer as a replacement (**C**).

All Cer subclasses, including the ultralong acylCer, are released from GlcCer by β-glucocerebrosidase (GlcCer’ase; Cer β-glucosidase; Fig. 1A)^4^. The inhibition of GlcCer’ase induces GlcCer accumulation in the SC and the formation of immature SC lipid lamellae and decreased barrier function^5^. A hereditary deficiency of GlcCer’ase occurs in Gaucher disease. A subset of type 2 Gaucher disease patients display ichthyosiform skin with a nearly four times higher epidermal GlcCer/Cer ratio and incompletely processed, loosely packed lipids throughout the SC^6^. Type 2 Gaucher mice exhibit similar features along with elevated transepidermal water loss (TEWL)^7^. Both Gaucher patients and mice have less Cer (both free and protein-bound) than healthy controls^6–8^.

Furthermore, both GlcCer’ase and sphingomyelinase require activation by saposins, *i*.*e*., sphingolipid activator proteins. Elimination of the saposin precursor prosaposin in a mouse model resulted in GlcCer accumulation and Cer decreases, alterations in protein-bound lipids and a striking abnormality in SC membrane maturation^9^. Decreased levels of prosaposin were detected in the skin of patients with psoriasis vulgaris^10^ and atopic dermatitis^11^. Alessandrini *et al*. further found decreased GlcCer’ase in non-lesional psoriatic skin compared to normal skin^12^.

The molecular basis for the detrimental effects of the retained glucose moiety in Cer on the SC lipid microstructure and permeability is not clear. Holleran *et al*. suggested that the persistence of GlcCer, rather than diminished Cer, is more likely to be the principal cause of the membrane structural abnormalities leading to the skin lesions in type 2 Gaucher disease^7^ because topical Cer did not prevent the deleterious effects of the GlcCer’ase inhibitor^5^. However, the inhibitor did not decrease Cer levels^5^.

The aim of our study was to prepare skin lipid membranes that simulate deficient GlcCer’ase to explore the molecular basis underlying the difference between the effects of a GlcCer accumulation and/or Cer decrease on the membrane microstructure and permeability and also the effect of topical GlcCer on human skin barrier. The control model membranes contained all major barrier lipids; the Cer fraction was gradually replaced by GlcCer in the models of GlcCer’ase deficiency (Fig. 1B,C). First, we prepared models with *N*-tetracosanoyl D-*erythro*-sphingosine (CerNS), which was then replaced by isolated human SC Cer (hCer; Fig. 1D). To compare the effect of GlcCer accumulation *versus* Cer deficiency, a series of membranes with diminished Cer without GlcCer were examined. The membrane permeabilities were evaluated using four markers: TEWL, electrical impedance, and the flux values of theophylline (TH) and indomethacin (IND). The effects of GlcCer/Cer on the lipid lamellar phases, chain order, and lateral packing were studied using X-ray powder diffraction (XRPD) and Fourier transform infrared spectroscopy (FTIR).

## Results

### Effect of GlcCer Accumulation on the Permeability of CerNS Model Membranes

First, we prepared samples containing CerNS/FFA/Chol. Solutions of each lipid were mixed in a 1:1:1 molar ratio, with 5 wt% CholS. To simulate defects in GlcCer processing, CerNS was subsequently replaced by GlcCer. To create the membrane, prepared lipid mixtures were sprayed on a supporting filter and annealed at 90 °C. The membrane permeabilities were evaluated using four markers: the flux of TH (a small molecule with balanced lipophilicity); the flux of IND (a large lipophilic molecule); electrical impedance (membrane opposition to electrical current) and TEWL. In these simple membranes, all the permeability markers except for TEWL indicated that the presence of GlcCer decreases the permeability (Supplementary Fig. S1). This finding is in contrast to the immature skin lipid barrier and increased TEWL in GlcCer’ase deficiency^7^. We assumed that these results might have been caused by the simplicity of the used models (only with one Cer subclass, CerNS).

### Effects of GlcCer Accumulation on the Permeability of Human Cer Membranes

**Figure 2:**
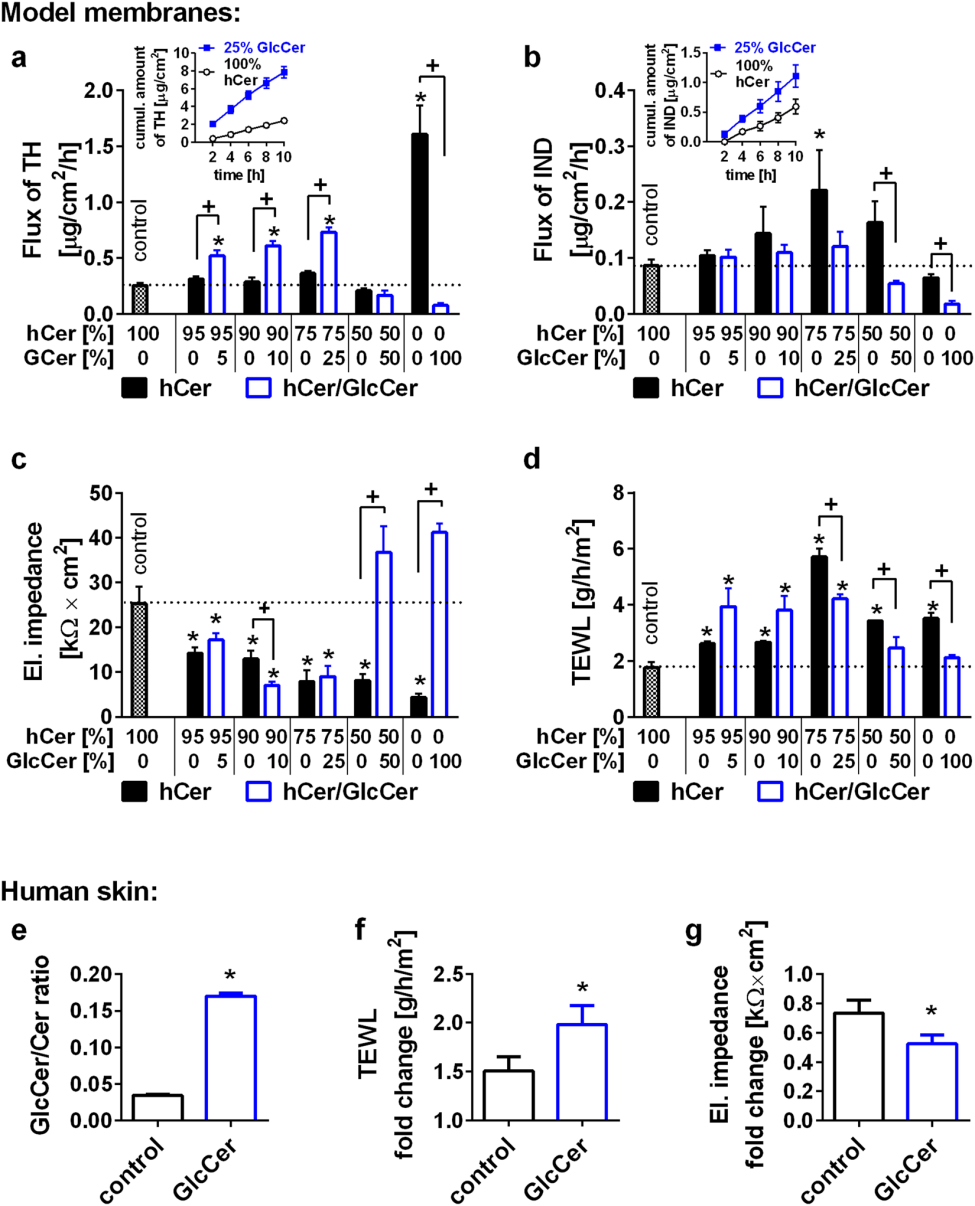
Permeabilities of the hCer Membrane Models (**a**–**d**) and Effects of Topical GlcCer on the Human Skin Barrier (**e–g**). Control membranes contained hCer/FFA/Chol/CholS; the disease models simulated accumulated GlcCer (blue) and/or diminished hCer (black) as indicated by the x-axes (molar %). The membrane permeabilities were studied using the flux of theophylline (TH; **a**) and indomethacin (IND; **b**), the electrical impedance (**c**) and the water loss (TEWL; **d**). The inserts in (**a**,**b**) show examples of the permeation profiles. Panel e shows the increase in the GlcCer/Cer ratio in the human SC after topical GlcCer application; panels f-g give the fold change in the TEWL and electrical impedance, respectively, induced by topical GlcCer or vehicle (control). Mean ± SEM, *n* = 4 (**a**–**d**) or 6 (**e**,**f**). *Significant difference compared with control at p < 0.05; +Significant difference between the membranes with and without GlcCer at p < 0.05.

To prepare a membrane model mimicking the SC lipid heterogeneity more closely, we isolated human SC Cer (hCer). High-performance thin layer chromatography (HPTLC) confirmed that the isolated Cer fraction contained all Cer subclasses in the correct proportions, including acylCer (Fig. 1D). The control membranes contained hCer, whereas in the disease models, hCer were subsequently replaced by 5–100% GlcCer (Fig. 2a–d, blue bars).

The flux of TH through the control membrane was 0.25 ± 0.03 µg/cm^2^/h. The replacement of 5–25% hCer by GlcCer increased the membrane permeability 2- to 3-fold over that of the control. Further replacement of hCer by GlcCer (by 50–100%) decreased the TH permeabilities below that of the control. The permeabilities to IND of the membranes with 5–25% GlcCer slightly (insignificantly) increased over that of the control (0.09 ± 0.01 µg/cm^2^/h) but decreased with higher GlcCer content. The electrical impedance of the control membrane containing hCer was 25 ± 4 kΩ × cm^2^. After the replacement of 5–25% hCer by GlcCer, the impedance decreased to 7–17 kΩ × cm^2^, which indicates a disturbed barrier, whereas further increases in GlcCer/hCer ratio increased the impedance. The TEWL of the control membrane was 1.8 ± 0.2 g/h/m^2^. All GlcCer membranes showed higher TEWL than the control with a bell-shaped concentration dependence with maxima at 5–25% GlcCer (3.9–4.2 g/h/m^2^).

Altogether, the model membranes containing 5–25% GlcCer displayed disrupted barrier function, including increased permeability to model compounds, decreased electrical impedance and increased TEWL. With the further substitution of hCer by GlcCer (by 50 and 100%), we found a trend towards improvement of the barrier properties of these model membranes.

### Effects of Diminished Cer without GlcCer Accumulation on the Permeability of Human Cer Membranes

To distinguish the contribution of increased GlcCer from that of decreased hCer content, we prepared a set of membranes with the same concentrations of hCer, FFA, Chol and CholS as in the latter experiment but without the addition of GlcCer (Fig. 2a–d, black bars). The TH flux was not significantly affected by the reduction of hCer content (0.21–0.31 µg/cm^2^/h), except for the total elimination of hCer, which led to a 6-fold higher permeability (1.60 ± 0.26 µg/cm^2^/h) over that of the control. In contrast, the IND flux increased with the hCer reduction to 75% (2-fold) and then decreased. The electrical impedance decreased with lower hCer content in all membranes compared to the control. The TEWL was significantly higher in all membranes with reduced hCer content than in the control with the maximum occurring for 75% hCer (5.7 ± 0.3 g/h/m^2^).

Thus, for 5–10% replacement of hCer by GlcCer, the presence of GlcCer appears to be a stronger contributor to the altered permeability than a lack of hCer. As hCer decreases, the lack of hCer disturbs the barrier, whereas GlcCer have no negative effects on permeability compared to that of the control.

### Permeability of Human Skin after Topical Application of GlcCer

**Figure 3:**
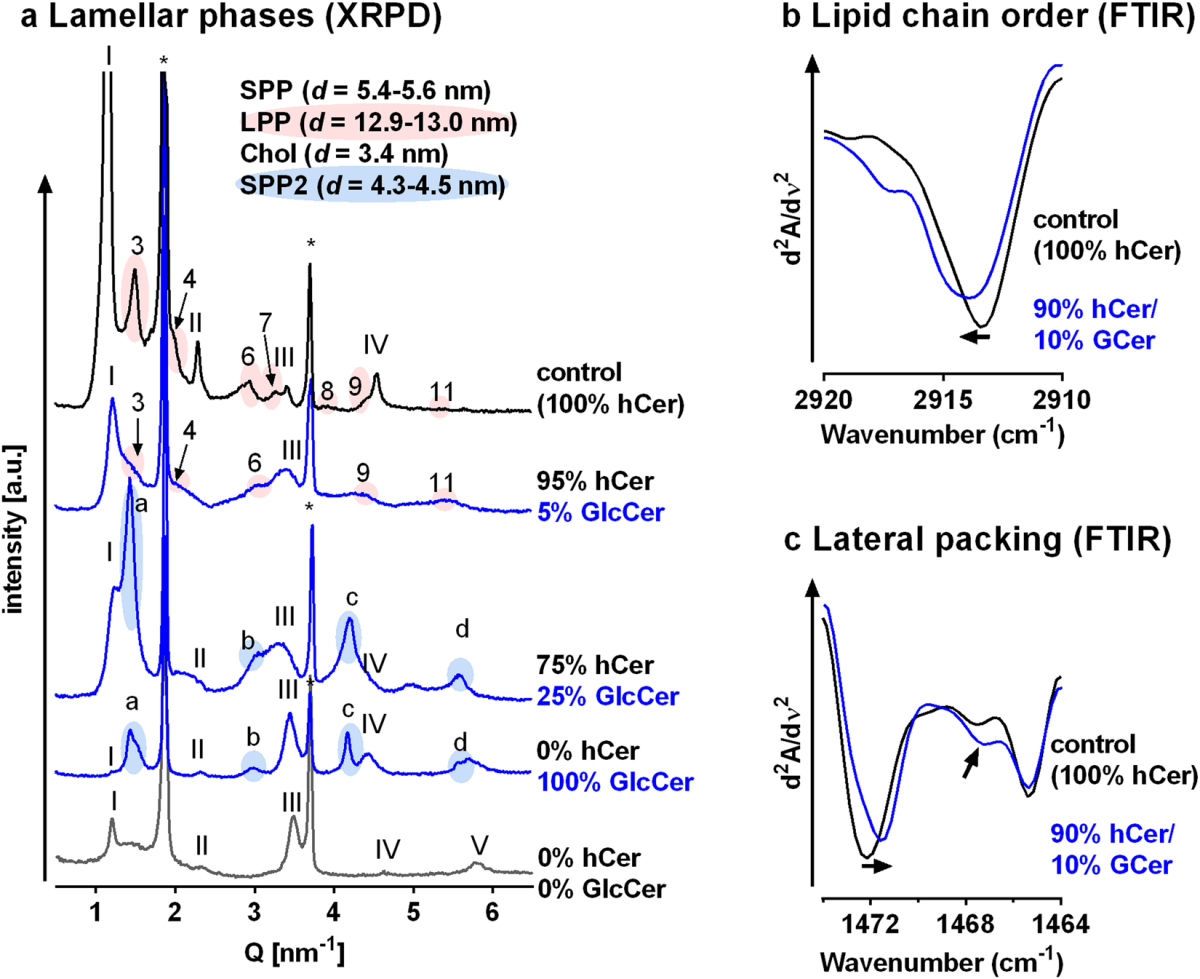
Lamellae phases, lipid chain order and lateral packing of selected hCer/GlcCer/FFA/Chol/CholS membranes studied using X-ray powder diffraction (XRPD; **a**) and Fourier transform infrared spectroscopy (FTIR; **b** and **c**). Roman numerals mark the short periodicity phase (SPP); Arabic numerals mark the long periodicity phase (LPP); asterisks mark crystalline cholesterol (Chol) reflections; and letters mark the additional short periodicity lamellar phase SPP2. The data in panels b and c are shown as second derivative spectra for clarity, and the arrows indicate the GlcCer-induced changes.

The permeability results from model membranes were verified using human skin (Fig. 2e–g). Topical application of GlcCer in propylene glycol/ethanol 7:3 (v/v) increased the SC GlcCer/Cer ratio 4.5-fold, as shown by HPTLC analysis. This amount of exogenous GlcCer in the human SC increased the TEWL almost 2-fold (from 5.8 ± 1.0 g/h/m^2^ before treatment to 10.8 ± 1.5 g/h/m^2^), a significantly greater effect than that of the vehicle (Fig. 3e). Thus, topical application of GlcCer on intact human skin disturbs the permeability barrier to water and partly mimics GlcCer’ase deficiency. The electrical impedance after the GlcCer application (52% of the pre-treatment value; Fig. 3g) confirmed a barrier impairment.

### Microstructure of the Membranes with Accumulated GlcCer and/or Diminished hCer

The microstructure of the hCer model membranes was studied by XRPD (Fig. 3a and Supplementary Fig. S3). All diffractograms contained two reflections corresponding to stacked Chol bilayers with a repeat distance *d* = 3.42 nm. The diffractograms of the control sample also contained a lamellar phase with *d* = 5.49 nm, which is analogous to the short periodicity phase (SPP) in the human SC, and a lamellar phase with *d* = 12.95 nm, which corresponds to the long periodicity phase (LPP). The LPP is critical for the human SC barrier^13–16^.

Upon the replacement of 5–10% hCer by GlcCer, we found Chol, SPP (*d* = 5.42–5.53 nm) and LPP (*d* = 12.85–12.86 nm), but the SPP and LPP reflections were weaker than those in the control membrane (Supplementary Fig. S3). When the GlcCer content was further increased to 25 and 50%, SPP (*d* = 5.41–5.42 nm) and Chol were found. The LPP was detected only in some samples with 25% GlcCer and disappeared for 50% GlcCer. In these membranes, we found an additional lamellar phase, SPP2, with *d* values of 4.51–4.56 nm. The sample in which all hCer were replaced by GlcCer showed Chol, SPP (*d* = 5.54 nm) and SPP2 lamellar phases (*d* = 4.38 nm).

The reduction of the hCer content to 95–75% (without GlcCer) did not change the lamellar phases compared to those of the control (Supplementary Fig. S2–S3). The LPP started to disappear at 50% hCer, while the SPP was apparent even in the complete absence of hCer (Fig. 3a).

Because the membrane with 10% GlcCer did not show any dramatic changes in microstructure (except for weaker SPP and LPP) but did undergo significant changes in permeability compared to that of the control, we further examined this membrane using FTIR (Fig. 3b,c). The presence of 10% GlcCer shifted the methylene stretching vibrations to higher wavenumbers and broadened these bands compared to those of the control, suggesting lipid chain disordering^17, 18^. The control membrane displayed orthorhombic lipid packing as indicated by a methylene scissoring doublet at 1472 and 1465 cm^−1^ ^18^. Exchange of 10% hCer for GlcCer slightly decreased the width of this doublet and increased the relative intensity of the middle component (at approximately 1467 cm^−1^), which corresponds to hexagonal lipid packing^18^. These changes indicate lipid disorder and less tight lipid packing.

## Discussion

Insufficient GlcCer’ase function in the skin leads to decreased Cer levels, structural defects in SC lipid lamellae and abnormal barrier function^5, 19^. Because the molecular basis for this impairment is not clear, investigations involving model lipid membranes should help to define the mechanisms underlying these barrier defects. Model membranes lack corneocytes but reproduce many important features of SC lipids, *e*.*g*., conformation, lateral packing, lamellar phases and permeability^13, 20–23^.

First, we investigated models based on a single Cer subclass – CerNS. However, replacement of CerNS replacement by GlcCer failed to mimic the barrier abnormality associated with GlcCer deficiency. Such failure of simple membranes was reported in models of sphingomyelinase deficiency^21^ and in models with acylCer^20^. This simplification of a skin lipid barrier may be advantageous in studies focused on structure-permeability relationships, but it may give misleading data in disease models. Thus, more complex lipid systems using Cer isolated from the human SC were prepared. The permeabilities of the control hCer model were close to the permeabilities of human skin (flux of TH 0.51 ± 0.01 µg/cm^2^/h, impedance 22 ± 1 kΩ × cm^2^ and TEWL 3–6 g/m^2^/h)^24^.

The accumulation of 5–25% GlcCer (with the concomitant loss of 5–25% hCer) significantly increased the TEWL and permeability to TH and decreased the electrical impedance (2–3.5-fold changes), whereas the permeability to the large and lipophilic IND remained unchanged. With a minor impairment of GlcCer-to-Cer processing, GlcCer appear to be the principal cause of barrier alterations, as suggested previously^7^. To validate our hCer membrane model, we tried to reproduce the effects of GlcCer accumulation in human skin. Topical application of GlcCer to intact human skin increased the SC GlcCer/Cer ratio by 4.5-fold. This artificial GlcCer accumulation in the skin increased the TEWL and decreased the impedance in a comparable manner to membrane models with a similar GlcCer/Cer ratio (10–25% GlcCer).

These levels of GlcCer accumulation and TEWL in both the membrane models and GlcCer-treated skin are relevant to *in vivo* findings. In UVB-irradiated mice, a 2.5-fold higher TEWL and a more than 3-fold increase in SC GlcCer over control values were found^25^. In Gaucher mice, a 10- to 15-fold elevation in GlcCer led to a 10- to 50-fold increase in the TEWL^7^; however, in type 2 Gaucher patients, the epidermal GlcCer/Cer ratio was only 3.9-fold higher than that of the normal epidermis^6^. In psoriasis, where GlcCer’ase levels are also decreased^12^, the TEWL was increased by 47% in uninvolved skin and by 2.7-fold in scaling psoriatic plaques compared to healthy skin^26^.

At 10% GlcCer, the lamellar phases were similar to control but with decreased periodicities and intensities of the SPP and LPP; and FTIR revealed decreased lipid chain order and less tight lipid packing compared to those of the control. At 25% GlcCer, the LPP was very weak and discernible only in some samples, while a new lamellar phase with 4.5 nm periodicity emerged. This finding is in good agreement with the permeability data because the LPP is indispensable for the skin barrier^27^. The nature of these changes corresponds to the structural defects upon GlcCer’ase deficiency observed by electron microscopy^5, 6, 9^. Disturbed lipid chain order and packing also occur in reconstructed human skin models, which have increased GlcCer levels in the SC compared to that of human skin^28^.

Further decrease of hCer to 50% or their complete absence led to profound differences between models with and without GlcCer. The loss of hCer (without the accumulation of GlcCer) led to 2- to 6-fold increased membrane permeability compared to that of the control (except for IND). XRPD of these hCer-deficient membranes showed SPP with Chol. The lack of LPP and the generally increased permeability associated with a 50% reduction in hCer might be related to a critical decrease in acylCer (*e*.*g*., Cer EOS), which are essential components of the skin barrier^27, 29^.

In contrast, replacement of 50–100% hCer with GlcCer resulted in a surprisingly good barrier –similar (TEWL) or slightly better than that of the control (impedance, TH, and IND). XRPD showed a SPP and Chol similar to those of hCer-deficient membranes but also an additional lamellar phase (SPP2) with 4.3–4.5 nm periodicity. This lamellar phase has not been reported *in vivo*, but GlcCer’ase deficient skin was not studied by XRPD. SPP2 was also present in the membrane in which hCer were completely substituted by GlcCer. Hence, we assumed that SPP2 might correspond to a GlcCer-enriched phase. Because this lamellar phase is the major difference between the hCer-depleted membranes with or without GlcCer, SPP2 might be the cause of the relatively good permeability barrier of the 100% GlcCer membranes. This assumption is supported by FTIR data that revealed well-ordered lipids with large orthorhombic domains in the 100% GlcCer membrane (Supplementary Fig. S4).

This GlcCer-based membrane, which effectively limited permeation of TH, IND, and ions, appears to be consistent with the lipid barrier in marine mammals, which mostly contains GlcCer^30^. Although marine mammals do not need as tight of a water barrier as terrestrial mammals, their epidermis also has to prevent the efflux of body components and the absorption of exogenous substances.

However, these results on model membranes with complete replacement of hCer by GlcCer are in strong contrast with data from Gaucher mice, which have a largely diminished water barrier^7^. This discrepancy may be explained by the lack of the corneocyte lipid envelope (CLE), *i*.*e*., lipids covalently attached to the corneocyte surface, in our models. The CLE is formed by ultralong ω-hydroxyGlcCer, which are attached to involucrin via their ω-hydroxyl, and, subsequently, glucose is removed by GlcCer’ase^8, 27^. The CLE provides a stable scaffold for the organization of the extracellular lipid matrix^31^. Lipid analysis of type 2 Gaucher mice showed up to 35-fold more GlcCer and 10-fold less Cer and FFA in the CLE compared to those of the normal control^8^. Increased GlcCer and decreased Cer and FFA were also found in the CLE of prosaposin-deficient mice^9^. The glucose residue in CLE lipids, which face the extracellular domains, could interfere with the organization of free SC lipids into compact lamellar membranes^3^. Because of the lack of barrier impairment in models with 100% GlcCer, we speculate that the major cause of the altered permeability barrier in severe GlcCer’ase deficiency would be the glucosylated CLE rather than the accumulation of free GlcCer.

In conclusion, we prepared model membranes simulating impaired GlcCer-to-Cer processing. Relatively minor impairment to the Cer generation from GlcCer diminished the permeability of the model and altered the membrane microstructure, in agreement with the effects of topical GlcCer in human skin and literature data. However, replacement of 50–100% human Cer by GlcCer maintained a rather good barrier to the four studied permeability markers. Although it is not possible to directly translate our results that were obtained using model lipid membranes to the *in vivo* skin barrier, these findings strongly suggest that the major cause of the impaired water permeability barrier in complete GlcCer’ase deficiency is not the inability of free GlcCer to form a competent barrier. We hypothesize that the most likely cause for the altered SC barrier is the retention of GlcCer bound in the CLE.

## Methods

### Isolation of Human Skin Cer (hCer)

The skin was obtained from female patients who had undergone abdominal plastic surgery. Informed consent has been obtained. The procedure was approved by the Ethics Committee of Sanus First Private Surgical Centre, Czech Republic and conducted according to the principles of the Declaration of Helsinki. The epidermis was heat separated from dermis at 60 °C. The SC was pooled from 6 donors (all female), isolated by trypsin treatment^32^, the lipids were extracted^33^ and purified by column chromatography^21^. The composition of hCer was verified and quantified using HPTLC^28, 34^. This Cer composition and average chain lengths of the individual Cer subclasses^35^ gave the molecular mass of hCer of 685 g/mol.

### Preparation of Model SC Membranes

FFA were mixed in a molar % that corresponds to the composition of human skin FFA^36^ (see Supplementary information). Then the FFA mixture was combined with equimolar amounts of Chol and sphingolipids (CerNS, hCer or GlcCer, for details, see Fig. 1 and Supplementary information) and 5 wt% of CholS. The lipid mixtures were dissolved in hexane/96% ethanol 2:1 (v/v) at concentration 4.5 mg/ml. Mixtures containing hCer created fine suspensions, which were homogenized by ultrasound. Then, the lipid solutions/suspensions (3 × 100 µl; 1.35 mg/cm^2^) were sprayed to Nuclepore polycarbonate filters with 15 nm porosity (Whatman, Kent, UK) under nitrogen using a Linomat V (Camag, Muttenz, Switzerland) equipped with additional y-axis movement^36^. Thus, each membrane (0.79 cm^2^) contained 1 mg of lipids. The prepared lipid membranes were dried in vacuum over P_4_O_10_ and solid paraffin and stored at −20 °C. The day before the permeation experiments, the lipid membranes were annealed at 90 °C for 10 min and then slowly (3–4 h) cooled to 32 °C. During this process, a lamellar structure was created. Afterward, the membranes were equilibrated at 32 °C and 45 ± 5% relative humidity for at least 24 h. The lipid films for FTIR experiments were prepared in the same manner. The homogeneity of the membranes was previously validated^37^.

### Membrane and Skin Permeability

#### Membrane permeability

The membranes were sandwiched between Teflon holders with an available diffusion area of 0.5 cm^2^ and mounted in the Franz diffusion cells with the lipid film facing the upper (donor) compartment. The bottom (acceptor) compartment was filled with PBS at pH 7.4 containing 50 mg/l gentamicin. The precise volume of the buffer (6.5 ± 0.1 ml) was individually measured for each cell and was included in the calculation of flux. The acceptor phase was stirred at 32 °C throughout the experiment. After a 12-h equilibration at 32 °C, the water loss through the membrane and electrical impedance were measured (see below).

Then, membranes received 100 µl of either 5% theophylline (TH) or 2% indomethacin (IND) in 60% propylene glycol and the cells were stirred at 32 °C. Propylene glycol had no adverse effects on the membranes^22, 37, 38^. The concentrations were selected so that all samples were saturated with the pertinent model drug to maintain the same thermodynamic activity throughout the experiment. This setup ensured sink conditions for the selected compounds. Samples of acceptor phase (300 µl) were withdrawn every 2 h over 10 h and were replaced by the same volume of PBS. During this period steady state situation was reached. The solubilities of TH and IND in the acceptor phase were 7.65 ± 0.02 mg/ml^24^ and 0.65 ± 0.03 mg/ml.

#### Skin permeability

The skin permeation experiments were performed using Franz diffusion cells with an available diffusion area of 1 cm^2^. The skin fragments were slowly thawed immediately before use, cut into squares approximately 2 × 2 cm and mounted into the diffusion cells. The acceptor compartments of the cells (16.5 ± 1.5 ml) were filled with PBS. The diffusion cells were stirred at 32 °C. After a 12-h equilibration, TEWL and electrical impedance were measured (see below). Afterward, 100 µl of 1% suspension of GlcCer in propylene glycol/ethanol 7:3 (v/v) or 100 µl of the vehicle solvent without lipids was applied on the skin. The diffusion cells were incubated at 32 °C. After 14 h, the donor samples were carefully removed using cotton swabs, the skin surface was rinsed with PBS and dried. After 2 h, TEWL and electrical impedance were measured (data are presented as a fold change after/before the treatment). At the end of these experiments, the diffusion cells were dismounted and the treated skin area was punched out. SC was isolated by trypsin treatment^32^ and the SC lipids were extracted^21^ and analysed by HPTLC^28, 34^.

### Transepidermal/Transmembrane Water Loss (TEWL)

TEWL was measured in the Franz cells with their upper parts removed using a Tewameter TM 300 probe and Multi-Probe Adapter Cutometer MPA 580 (CK electronic GmbH, Köln, Germany) at 34–38% relative air humidity and 22–24 °C. The measured TEWL was corrected for the diffusion cell setup^37^.

### Electrical Impedance

The donor compartment received 500 µl of PBS at pH 7.4 for 30 min. The impedance was measured using an LCR meter 4080 (Conrad Electronic, Hirschau, Germany) by immersing one electrode in the donor compartment and the second in the acceptor compartment of the cell^22, 37, 38^.

### X-Ray Powder Diffraction (XRPD) and Fourier Transform Infrared Spectroscopy (FTIR)

The lipid mixtures were prepared in the same manner as those for permeation experiment, but lipid mixtures were sprayed onto a cover glass instead of polycarbonate filters. Samples were equilibrated and hydrated at 100% relative humidity for 12 h. For more details see refs 20 and 38 and Supplementary information.

### Data treatment

The cumulative amounts of TH and IND that penetrated through the lipid membrane were corrected for the acceptor phase replacement and exact acceptor volume of the diffusion cells and were plotted against time. The steady state flux of TH or IND [µg/cm^2^/h] was calculated as a slope of the linear regression function obtained by fitting the linear region of the plot in Excel. The data are presented as the means ± standard error of the mean (SEM), and the number of replicates is given in the pertinent figure. Two groups of data were compared using unpaired t-tests; for comparison of three or more groups, one-way analysis of variance with Dunnett’s post hoc test method was used and p < 0.05 was considered significant.

## Electronic supplementary material

Supplementary material
