# Supplementary material for: Permeability Barrier and Microstructure of Skin Lipid Membrane Models of Impaired Glucosylceramide Processing
Source: Sci Rep. 2017 Jul 25;7:6470. doi: 10.1038/s41598-017-06990-7 (PMC5527096; doi:10.1038/s41598-017-06990-7)
Supplement: Supplementary file 1 — Supplementary material [file 41598_2017_6990_MOESM1_ESM.pdf]

SUPPLEMENTARY INFORMATION

Permeability Barrier and Microstructure of Skin Lipid Membrane Models of Impaired Glucosylceramide Processing

Michaela Sochorová[1], Klára Staňková[1], Petra Pullmannová[1], Andrej Kováčik[1], Jarmila Zbytovská[2,3], Kateřina Vávrová[1,*]

[1]Skin Barrier Research Group, Charles University, Faculty of Pharmacy, Hradec Králové, 500 05, Czech Republic;

[2]Department of Pharmaceutical Technology, Faculty of Pharmacy, Hradec Králové, 500 05, Czech Republic

[3]Department of Organic Technology, University of Chemistry and Technology Prague, 166 28, Czech Republic

* katerina.vavrova@faf.cuni.cz

Supplementary figures

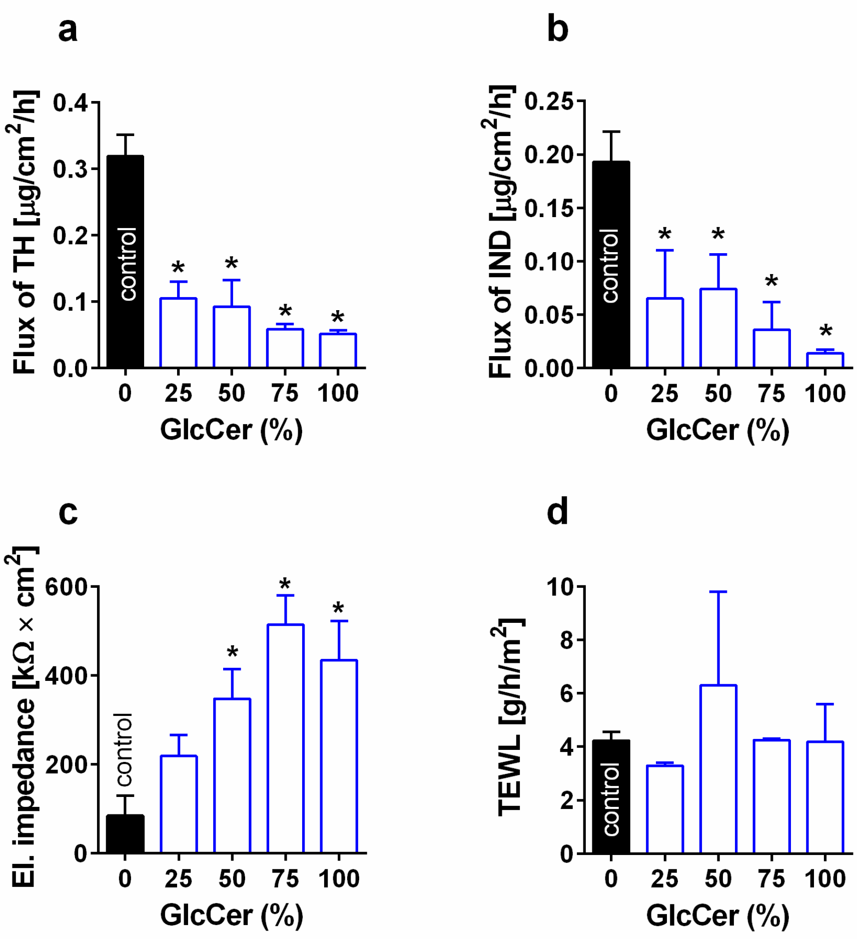

Supplementary Figure S1. Permeabilities of the Cer NS membrane models composed of 1:1:1 molar sphingolipid (CerNS and/or GlcCer)/FFA/Chol and 5 wt% CholS: flux of theophylline (TH; panel a), flux of indomethacin (IND; panel b), electrical impedance (panel c) and water loss (TEWL; panel d). The x-axes show the molar % of GlcCer from the sphingolipid fraction (Cer+GlcCer) of the membrane. Mean±SEM, n = 3-12. * Significant difference compared with control at $p < 0.05$.

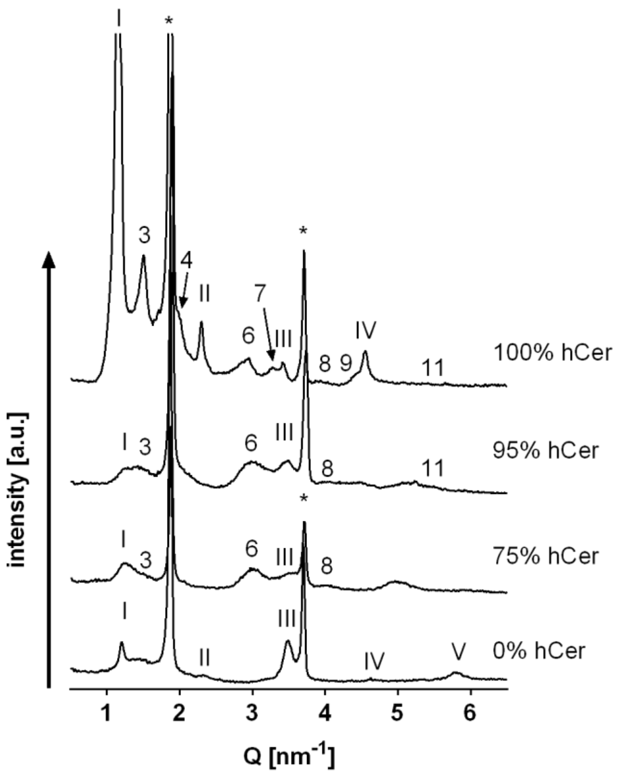

Supplementary Figure S2. Lamellar phases of selected hCer/FFA/Chol/CholS membranes studied using X-ray powder diffraction (XRPD). Roman numerals mark the short periodicity phase (SPP); Arabic numerals mark the long periodicity phase (LPP); and asterisks mark crystalline cholesterol (Chol).

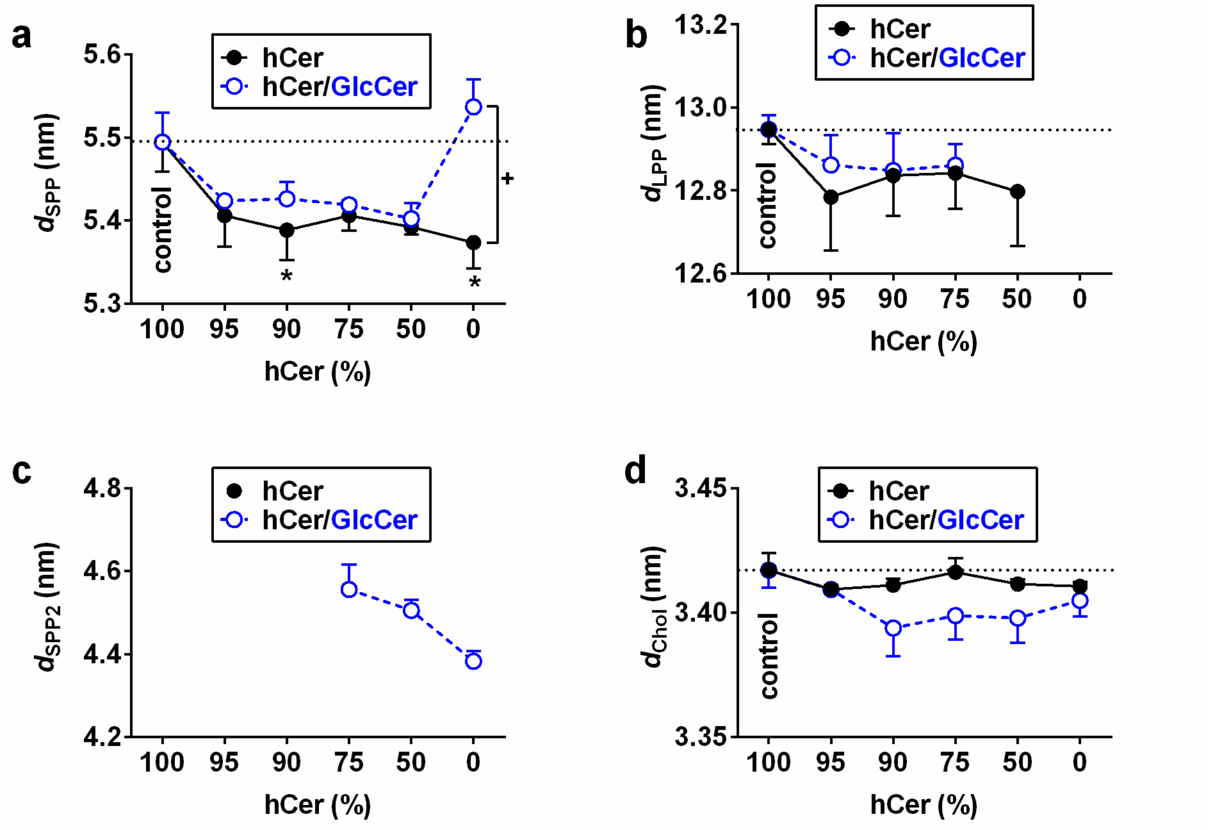

Supplementary Figure S3. Repeat distances (*d*) of the lamellar phases in the hCer/GlcCer/FFA/Chol/CholS membranes studied by X-ray powder diffraction (XRPD). Panels a-d show the *d* values of SPP, LPP, SPP2, and Chol, respectively. Mean±SEM, $n = 2$-6. * Significant difference compared with control at $p < 0.05$; + significant difference between the membranes with and without GlcCer at $p < 0.05$.

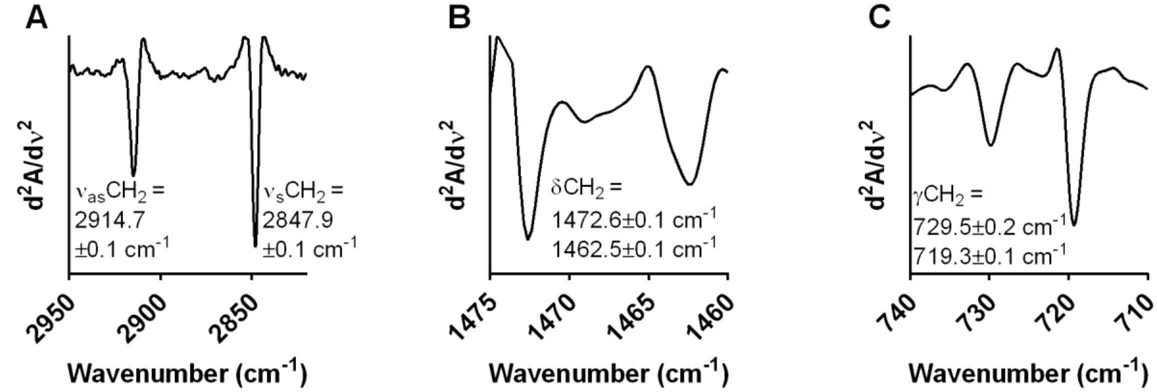

Supplementary Figure S4. Infrared spectra of the GlcCer/FFA/Chol/CholS membrane. Panel A shows methylene asymmetric and symmetric stretching vibrations that suggest well-ordered lipid chains. Panels B and C show methylene scissoring and rocking doublets, respectively, that indicate orthorhombic chain packing. All data are shown as second derivative spectra for clarity.

Supplementary materials and methods

Chemicals

N-tetracosanoyl-D-*erythro*-sphingosine (CerNS) and D-glucosyl-β1-1'-*N*-stearoyl-D-*erythro*-sphingosine (GlcCer) were purchased from Avanti Polar Lipids (Alabaster, AL, USA). Cholesterol from lanolin (Chol), sodium cholesterol sulfate (CholS), hexadecanoic acid, octadecanoic acid, eicosanoic acid, docosanoic acid, tetracosanoic acid, theophylline anhydrous (TH), indomethacin (IND) and other chemicals were from Sigma-Aldrich (Schnelldorf, Germany).

Human skin

The skin was obtained from female patients who had undergone abdominal plastic surgery in Sanus surgical center (Hradec Králové, Czech Republic). The procedure was approved by the Ethics Committee and conducted according to the principles of the Declaration of Helsinki. The residual subcutaneous fat was carefully removed from the skin fragment by a scalpel. The skin was washed in phosphate-buffered saline (PBS) at pH 7.4 with 50 mg/l gentamicin, blotted dry and stored at -20°C.

Isolation of human skin Cer (hCer)

The extracted human SC lipids (pooled from skin fragments from 6 subjects) were purified by column chromatography on Silica gel 60 (Merck, Darmstadt, Germany). The SC lipids classes were eluted sequentially with chloroform/acetic acid 99:1 (v/v) and then chloroform/methanol in 100:1, 50:1, 10:1, 3:1, 2:1, 1:1 and 1:2 ratios (v/v). The fractions containing the eluted Cer were collected, and the solvent was removed using a rotatory vacuum evaporator and then in high vacuum over P_4O_{10} and solid paraffin. The isolated hCer were stored under nitrogen at -20°C. The composition of the isolated hCer was verified using high-performance thin layer chromatography (HPTLC) [1,2].

Preparation of model SC membranes

First, mixture of FFA was prepared. Individual FFA were dissolved in hexane/96% ethanol 2:1 (v/v) and mixed in a molar % that corresponds to the composition of human skin FFA: 1.8 % hexadecanoic acid, 4.0 % octadecanoic acid, 7.6 % eicosanoic acid, 47.8 % docosanoic acid and 38.8 % tetracosanoic acid.[3] Chol, CerNS or hCer were dissolved in hexane/96 % ethanol 2:1 (v/v) (hCer created a fine suspension that was carefully homogenized before use[4]), GlcCer in hexane/96% ethanol 1:1 (v/v) and CholS in 96% ethanol.

The organic solutions/suspension were mixed in the desired ratio (FFA, Chol, CerNS or hCer in equimolar ratio with addition of 5 wt% of CholS = control sample). Lipid solutions for the preparation of model membranes with hCer replaced by GlcCer contained the same amount of FFA, Chol and CholS, only the "Cer fraction" (= CerNS or hCer with or without GlcCer) was modified (for details, see Figure 1). Lipid solutions for model membranes with reduced hCer content contained less lipids in Cer fractions, therefore total amounts of lipids were lower in these samples.

The solvents from sample mixtures were evaporated and samples were dried. Then the samples were redissolved in hexane/96% ethanol 2:1 (v/v) at 4.5 mg/ml. Nuclepore polycarbonate filters were washed in hexane/96% ethanol 2:1 (v/v), dried and mounted in steel holders with an opening of 1 cm diameter, which exposed 0.79 cm^2 of the filter. The lipid solutions were sprayed on the filters under a stream of nitrogen using Linomat V equipped with additional y-axis movement. On one filter (1 cm^2) were applied 300 µl of lipid solution (=1.35 mg lipids), divided in three steps with changing the direction of application after each step. Prepared lipid membranes were kept in the steel holders also during heating to 90 °C and subsequent cooling down. The membranes were directly transferred from the steel holders to Teflon holders used in permeation experiments.

High-performance liquid chromatography (HPLC)

The TH- and IND- containing samples of acceptor phase were measured by isocratic reverse-phase HPLC using s Shimadzu Prominence instrument (Shimadzu, Kyoto, Japan) consisting of LC-20AD pumps with a DGU-20A3 degasser, SIL-20A HT autosampler, CTO-20AC column oven, SPD-M20A diode array detector, and a CBM-20A communication module. Data were analyzed using the LCsolutions 1.22 software. Reverse phase separation of TH was achieved in a LiChroCART 250-4 column (LiChrospher 100 RP-18, 5 µm, Merck, Darmstadt, Germany) at 35°C using 4:6 methanol/0.1 M NaH_2PO_4 (v/v) as a mobile phase at a flow rate of 1.2 ml/min. Acceptor phase sample (20 µl) was injected into the column, and the UV absorption of the effluent was measured at 272 nm. The retention time of TH was 3.2±0.1 min. The IND samples were assayed on a LiChroCART 250-4 column (LiChrospher 100 RP-18, 5 µm, Merck) using a mobile phase containing 90:60:5 acetonitrile/water/acetic acid (v/v/v) at a flow rate of 2 ml/min. Next, 100 µl of acceptor phase sample was injected into the column, which was maintained at 40°C. The UV absorption was monitored at a wavelength of 260 nm, and the retention time of IND was 3.1±0.1 min. Both methods were previously validated according to EMA European Medicines Agency, Guideline on bioanalytical method validation (2011) [5].

X-ray powder diffraction (XRPD)

The XRPD data were collected at ambient temperature with an X´Pert PRO θ-θ powder diffractometer (PANalytical B.V., Almelo, Netherlands) with parafocusing Bragg-Brentano geometry using CuK_α radiation (λ = 1.5418 Å, U = 40 kV, I = 30 mA) in modified sample holders over the angular range of 0.6-30° (2θ). Data were scanned with an ultrafast detector X´Celerator with a step size of 0.0167° (2θ) and a counting time of 20.32 s $step^{-1}$. The data were evaluated using the software package HighScore Plus (PANalytical B.V., Almelo, Netherlands). The XRPD diffractograms show the scattered intensity as a function of the scattering vector Q [nm^{-1}], which is proportional to the scattering angle 2θ according to the equation: $Q = 4\pi\ sin\theta/\lambda$ (λ = 0.15418 nm is a wavelength of the X-rays). The repeat distance d [nm] characterizes the regular spacing of parallel lipid bilayers arranged on a one-dimensional lattice, a lamellar phase (L). The diffractograms of lamellae phases exhibit a set of Bragg reflections whose reciprocal spacing are in characteristic ratios of $Qn = 2\pi n/d$ (reflection´s order number n = 1, 2, 3…). The repeat distance

d was obtained from the slope a of a regression function of the dependence $Qn = a \times n$, according to the equation $d = 2\pi/a$.

Fourier transform infrared spectroscopy (FTIR)

Infrared spectra were collected on a Nicolet 6700 spectrometer (Thermo Scientific, USA) equipped with a single-reflection MIRacle ATR ZnSe crystal (PIKE technologies, Madison, USA). A clamping mechanism with constant pressure was used. The spectra were generated by the co-addition of 256 scans collected at a resolution of 2 cm^{-1} and analyzed using Bruker OPUS software.

References

1 Vavrova, K. *et al.* Filaggrin Deficiency Leads to Impaired Lipid Profile and Altered Acidification Pathways in a 3D Skin Construct. *J Invest Dermatol* **134**, 746-753, doi:10.1038/jid.2013.402 (2014).

2 Wallmeyer, L. *et al.* Stimulation of PPARα normalizes the skin lipid ratio and improves the skin barrier of normal and filaggrin deficient reconstructed skin. *J. Dermatol. Sci.* **80**, 102-110 (2015).

3 Groen, D., Gooris, G. S. & Bouwstra, J. A. Model Membranes Prepared with Ceramide EOS, Cholesterol and Free Fatty Acids Form a Unique Lamellar Phase. *Langmuir* **26**, 4168-4175, doi:10.1021/la9047038 (2010).

4 Pullmannová, P. *et al.* Effects of sphingomyelin/ceramide ratio on the permeability and microstructure of model stratum corneum lipid membranes. *Biochimica et Biophysica Acta (BBA) - Biomembranes* **1838**, 2115-2126, doi:https://doi.org/10.1016/j.bbamem.2014.05.001 (2014).

5 Školová, B., Kováčik, A., Tesař, O., Opálka, L. & Vávrová, K. Phytosphingosine, sphingosine and dihydrosphingosine ceramides in model skin lipid membranes: permeability and biophysics. *Biochim. Biophys. Acta-Biomembranes* **1859**, 824–834, doi:http://dx.doi.org/10.1016/j.bbamem.2017.01.019 (2017).
